# Evaluating *Paenibacillus odorifer* for its potential to reduce shelf life in reworked high-temperature, short-time fluid milk products

**DOI:** 10.3168/jdsc.2021-0168

**Published:** 2022-01-10

**Authors:** Casey E. Rush, Jared Johnson, Samantha Burroughs, Brandon Riesgaard, Alejandro Torres, Lisbeth Meunier-Goddik, Joy Waite-Cusic

**Affiliations:** Food Science and Technology, Oregon State University, Corvallis 97331

## Abstract

Rework is a common practice used in the dairy industry as a strategy to help minimize waste from processing steps or errors that might otherwise render the product unsaleable. Dairy processors may rework their high-temperature, short-time (HTST) fluid milk products up to code date (21 d) at a typical dilution rate of ≤20% rework into ≥80% fresh raw milk. Bacterial spores present in raw milk that can survive pasteurization and grow at refrigeration temperatures are often responsible for milk spoilage. However, the potential impact of growth and thermal resistance of organisms in reworked product has not been investigated. Our objective was to characterize growth, sporulation, and thermal resistance of *Paenibacillus odorifer* under conditions representative of extreme storage conditions (time and temperature) of reduced fat (2%) and chocolate milk to evaluate whether product containing rework would have a reduced shelf life. Commercial UHT-pasteurized 2% milk and chocolate milk were independently inoculated with 4 strains of *P. odorifer* at 1 to 2 log cfu/mL and stored at 4°C and 7°C for 30 d. Changes in *P. odorifer* cell densities were determined by standard serial dilution with spread plating on tryptic soy agar with yeast extract and incubation at 25°C for 48 h. Spore counts were determined following thermal treatment at 80°C for 12 min. Thermal resistance of a cocktail of *P. odorifer* in milk was determined after treatments at 63°C for 30 min and 72°C for 15 s. Strains of *P. odorifer* grew rapidly at 7°C and reached a maximum cell density of ~8 log cfu/g in both 2% and chocolate milk within 12 d. All strains grew more slowly at 4°C and had not reached maximum cell density by 21 d. With extreme temperature abuse (25°C, 24 h), *P. odorifer* will sporulate in milk; however, thermally resistant subpopulations, including spores, did not develop in milk at 4°C until after stationary phase was achieved (>24 d). Vegetative cells of *P. odorifer* were verified to be sensitive to pasteurization (>7 log reduction); therefore, *P. odorifer* would not be expected to contribute to reduced shelf life of fluid milk products containing rework, even with extended storage before rework.

## Highlights

• *Paenibacillus odorifer* does not sporulate within the shelf life of HTST (high-temperature, short-time) milk.
• The microbial quality of reworked milk products is unlikely to be affected by *P. odorifer.*
• We found evidence of a *P. odorifer* subpopulation surviving 72°C/15 s, but not 80°C/12 min.

## Graphical Abstract

**Figure:**
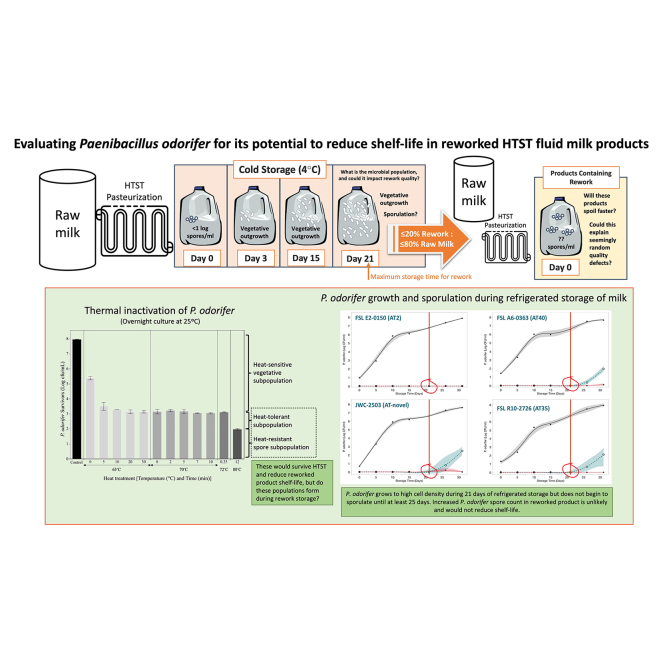
**Summary:** Rework practices used by the dairy industry have the potential to negatively influence product quality; however, this has not been previously investigated. The goal of this study was to use *Paenibacillus odorifer*, a problematic spore-forming spoilage bacterium, as the target organism to quantify growth rates, thermal resistance, and sporulation during the storage phase of product destined for rework. This study demonstrated the growth potential of *P. odorifer* during refrigerated storage of chocolate and unflavored milk and the inability for *P. odorifer* to sporulate in these products within their maximum shelf life and potential use in rework applications. Due to the heat sensitivity of *P. odorifer* vegetative cells, this study indicates that current rework practices are unlikely to lead to increased milk spoilage associated with this bacterium.

Quality defects and premature spoilage of pasteurized fluid milk is commonly caused by the growth of psychrotrophic microorganisms. There are 2 primary sources of spoilage organisms in fluid dairy products: heat-resistant psychrotrophic sporeformers from raw milk that survive the pasteurization process and psychrotrophic post-pasteurization contaminants (Alvarez, 2009; Trmčić et al., 2015; Martin et al., 2018). Post-pasteurization contaminants are commonly gram-negative bacteria present in the dairy processing environment that must be mitigated through sanitation programs (Gopal et al., 2015; Reichler et al., 2018). *Bacillus* and *Paenibacillus* spores are commonly found in raw milk and are collected during milking from the farm environment (Meer et al., 1991; Coorevits et al., 2008; Huck et al., 2008). These spores survive HTST pasteurization treatments (72°C, 15 s) and germinate and grow under refrigeration conditions (Washam et al., 1977; Collins, 1981; Huck et al., 2007). Sporogenesis is an adaptive characteristic triggered when a vegetative cell is placed in unfavorable environmental conditions (e.g., lack of nutrients, unfavorable storage temperature). When conditions are favorable, the spore can germinate into a vegetative cell, and replication then proceeds (Gauvry et al., 2017). *Paenibacillus* spp. are the dominant genus found at elevated levels (≥6 log cfu/mL) in pasteurized milk at the end of shelf life (Huck et al., 2008; Ranieri and Boor, 2009; Ivy et al., 2012). Quality defects have been reported to occur at cell densities >6.0 log cfu/mL for milk products contaminated with *Paenibacillus* spp., including off-flavors and textural changes resulting from proteolysis and lipolysis (Fromm and Boor, 2004; Alvarez, 2009; Ivy et al., 2012).

Management of microbial quality of milk is further complicated for dairy processors using reworking practices to minimize waste. According to the Dairy Practices Council, rework is defined as, “Clean, unadulterated food that has been removed from processing for reasons other than insanitary conditions or that has been successfully reconditioned by reprocessing and that is suitable for use as food” (Dairy Practices Council, 2005). The source of product to be reworked has commonly failed a quality standard, often due to underfilled containers, cosmetic packaging flaws, unsold product (products approaching sell-by date), elevated microbial counts, leakers, or diluted product reclaimed from the fillers between product changeovers and following clean-in-place (CIP) procedures. Dairy processors commingle these products with raw milk, resulting in a fresh product with a new code date. Common dilution rates used by the industry are 20% rework for unflavored milk and 10% rework for chocolate-flavored milk products (Rush et al., 2021). The timeframe for reworking HTST-pasteurized products typically falls between 14 and 22 d, with flavored products having a shorter shelf life (Ranieri and Boor, 2009). Products contaminated with psychrotrophic bacteria at the time of packaging can reach cell densities of >6.0 log cfu/mL within this timeframe (Douglas et al., 2000; Buehler et al., 2018; Beno et al., 2020). If these organisms survived pasteurization, they could have a negative effect on the shelf life of the newly processed product. Although there have been no prior investigations into microbiological quality implications of reworking milk products, processors report increased microbial cell densities and frequent flavor defects in reworked products compared with products that do not contain rework (Rush et al., 2021).

The majority (59%) of *Paenibacillus* spp. isolated from dairy products and dairy environments are *Paenibacillus odorifer* (Beno et al., 2020). Spores of *P. odorifer* germinate following pasteurization and replicate in pasteurized milk throughout its refrigerated shelf life (Beno et al., 2020). *Paenibacillus odorifer* has been demonstrated to grow in skim milk, whole milk, or skim milk broth (**SMB**) at 6°C and to reach final cell densities of >4 log cfu/mL within 14 d (Beno et al., 2020) and >6 log cfu/mL within 21 d (Ivy et al., 2012; Moreno Switt et al., 2014). The growth rate of *P. odorifer* in SMB has been modeled at 4°C (0.4 log cfu/mL per day); however, minimum growth temperature for *P. odorifer* is typically reported as 5°C (Berge et al., 2002).

There are multiple stages of fluid milk production where potential *P. odorifer* contamination, growth, and sporulation could pose an elevated risk in reworked product. First is the contamination level of *P. odorifer* spores in raw milk. Psychrotrophic spore density in fluid milk averages 1 spore/6 mL of raw milk (−0.79 log spores/mL; Buehler et al., 2018). Pasteurization will shock these spores to germinate in packaged product and grow during potential onsite storage of milk destined for rework. Growth behavior of *P. odorifer* in SMB at 6°C has been reported as an estimate of growth potential during distribution, retail, and consumer storage. However, dairy processing facilities typically have more consistent temperature control, and milk products held for rework are stored at ≤4°C. Growth of *P. odorifer* has been predicted, but not confirmed, at 4°C (Buehler et al., 2018). This information is necessary to determine the microbial burden of milk that will be diluted into fresh product and repasteurized. Although it is known that *P. odorifer* spores survive pasteurization, there is a lack of information on the heat resistance of vegetative cells and no evidence to indicate whether *P. odorifer* would sporulate in milk under these storage conditions. Martin et al. (2018) reported that a spoilage event can occur in a product container in which only 1 spore is present. If high levels of *P. odorifer* spores develop in milk products during the storage period before rework, then increased spoilage would be likely for milk products containing rework (Vissers et al., 2007; Buehler et al., 2018). Understanding the time and temperature conditions that lead to *P. odorifer* sporulation could guide dairy processors to limit the storage time of milk that might be reworked to minimize premature spoilage.

Our overall objective was to determine the potential effect of *P. odorifer* on milk products containing rework. This study was designed to characterize growth and behavior of *P. odorifer* in reduced fat (2%) and chocolate milk stored under conditions used by the dairy industry before reworking into fresh product. Growth rates, maximum cell density, and thermal resistance are critical parameters to determine the potential impact of *P. odorifer* on fluid milk products containing rework.

Strains of *P. odorifer* representing diverse *rpoB* allele types (AT) were used in this study: FSL A6-0363 (AT40), FSL R10-2726 (AT35), FSL E2-0150 (AT2), and JWC-2503 (AT1922). The first 2 strains were originally isolated from pasteurized milk and the latter 2 from pasteurized chocolate milk. Isolates were revived from frozen storage (−80°C) by transferring to tryptic soy broth with 0.3% yeast extract (TSBYE, Neogen) with incubation at 25°C for 24 h. Cultures were streaked for isolation on tryptic soy agar plates supplemented with 0.3% yeast extract (TSAYE, Neogen) and incubated at 25°C for 48 h. A single isolated colony of each strain was independently transferred into TSBYE and incubated at 25°C for 24 h. The resulting culture was serially diluted in 0.1% peptone water (Neogen) to a final cell density of 6 log cfu/mL, which served as the inoculum.

Ultra-high temperature pasteurized reduced fat (2%) fluid milk (Lactalis American Group) and lowfat (1%) chocolate milk (Nestlé USA Inc.) products were purchased at retail. Milk (1,500 mL) was aseptically transferred to Whirl-Pak bags (Nasco) and stomached for 30 s. Each bag was inoculated with a single *P. odorifer* strain to a final cell density of 1 to 2 log cfu/mL. Inoculated milk samples were aliquoted (200 mL) into sterile 237-mL plastic jars (Uline) in triplicate and incubated at 4°C and 7°C for 31 d. The cell density of *P. odorifer* was determined by standard serial dilution (0.1% peptone water) and spread plating on TSAYE with incubation at 25°C for 48 h. Growth rates were determined using JMP Pro v16 software (SAS Institute Inc.) by applying a line of fit to the exponential growth phase for each strain.

For thermal inactivation studies, each *P. odorifer* strain was transferred to 25 mL of reduced-fat milk and incubated at 25°C for 24 h. Cultured milk samples were combined and shaken for 30 s to create a *P. odorifer* cocktail and stored at 4°C for 24 h before use. Aliquots (900 μL) of reduced fat milk were transferred into microcentrifuge tubes and inoculated with 100 μL of *P. odorifer* cocktail to a cell density of 8 log cfu/mL. A dry heating block (Benchmark Dry Bath) was used to treat milk samples at 63°C for 0 to 30 min, 70°C for 0 to 10 min, 72°C for 15 s (HTST), and 80°C for 12 min (spore count). Thermal treatments were performed in triplicate. A thermocouple (EL-USB-TC-LCD, ThermoWorks) was held in an uninoculated milk sample to monitor temperature to establish come-up time. Heat-treated samples were immediately transferred to ice and survivors were enumerated using plating methods described above.

Growth of individual *P. odorifer* strains over the course of the 21-d shelf life of fluid milk products at 4°C and 7°C is shown in Figure 1. This information is relevant because the dairy industry may rework packaged product that has been stored under refrigerated conditions for 3 to 21 d before incorporating as rework into fresh product (Rush et al., 2021). All 4 strains were able to grow in both reduced fat and chocolate milk at both temperatures. Maximum growth rates over the first 7 to 10 d were similar between unflavored and flavored milk products for all strains at both temperatures (4°C = 0.39–0.53 log cfu/mL per day; 7°C = 0.83–0.98 log cfu/mL per day). Similar growth rates for *P. odorifer* in whole milk (7°C = 1.01 cfu/mL per day) and SMB (6°C = 0.6 cfu/mL per day) have been reported (Buehler et al., 2018; Sun et al., 2021); however, this is the first report of *P. odorifer* growing at temperatures below 5°C (Berge et al., 2002; Priest, 2015), as predicted by Buehler et al. (2018).

**Figure 1:**
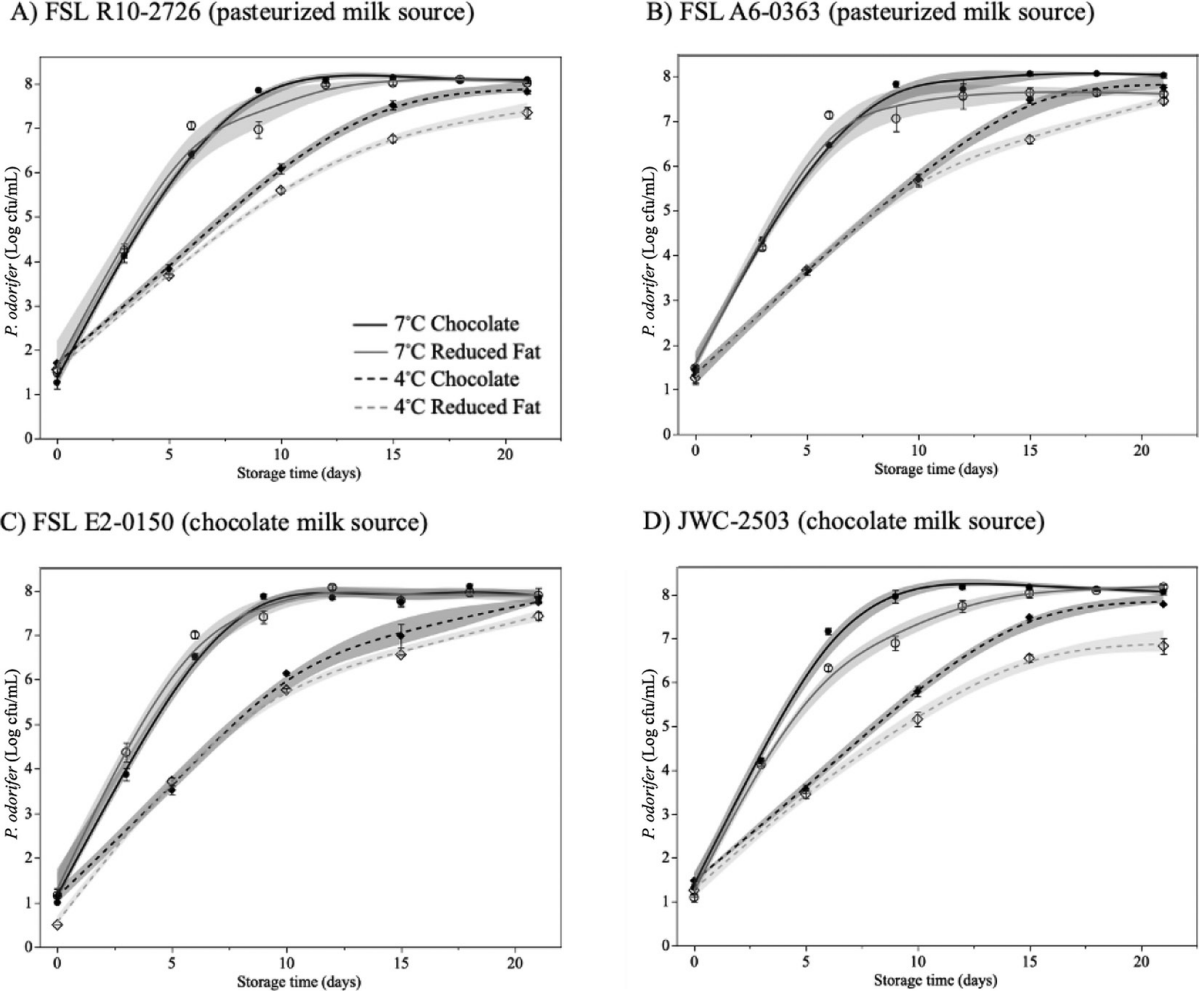
Growth behavior of *Paenibacillus odorifer* strains (A) FSL R10-2726, (B) FSL A6-0363, (C) FSL E2-0150, and (D) JWC-2503 in chocolate and reduced fat milk stored at 4°C and 7°C for 21 d. Data points indicate mean log cfu/mL (n = 3) ± standard error.

All strains demonstrated slower growth and a lower cell density after 21 d of storage at 4°C in unflavored milk (6.83–7.45 log cfu/mL) than in chocolate milk (7.74–7.83 log cfu/mL). This difference in growth was most obvious for *P. odorifer* JWC-2503 (Figure 1D). This strain was originally isolated from a spoiled chocolate milk product and demonstrated improved growth in chocolate milk compared with reduced-fat milk. *Paenibacillus odorifer* FSL E2-0150 was also originally isolated from chocolate milk but grew equally well in both products (Figure 1C). The observed increased growth rate and final cell density in chocolate milk may be attributed to the addition of sucrose, as increased growth of *P. odorifer* has been noted in media supplemented with sucrose (Priest, 2015). There is little discussion of chocolate milk spoilage in the published literature, with no specific information on the growth of *P. odorifer*. Faster spoilage rates of commercial chocolate milk products, including the lack of a lag phase compared with unflavored milk growth curves have been reported (Orleans, 2011). Increased growth rates of some strains of *P. odorifer* in chocolate milk may contribute to the reduced shelf life of chocolate milk reported by processors.

A primary goal of these experiments was to determine the maximum cell density of *P. odorifer* in milk after 21 d of storage in order to quantify the potential microbial contribution when mixed with raw milk for reprocessing. Previous research on fluid milk spoilage has focused on microbial growth at 6 to 7°C to mimic temperatures during distribution, at retail, and throughout storage in home refrigerators, whereas the storage temperature for milk held for rework is tightly controlled at 4°C. After 21 d of storage, *P. odorifer* ranged from 6.8 to 7.5 log cfu/mL at 4°C and 7.6 to 8.2 log cfu/mL at 7°C (Figure 1). The mean cell density of JWC-2503 at 4°C (6.8–0.2 cfu/mL) was lower than that of all other strains held at this temperature (7.4–7.5 cfu/mL).

Thermal inactivation experiments were performed to estimate the efficacy of pasteurization to reduce high cell density populations (>7 log cfu/mL) of *P. odorifer* present in milk destined for rework (Figure 2). Initial *P. odorifer* cell density in milk subjected to thermal treatments was 7.9 log cfu/mL. As milk was heated to 63°C (come-up time), the count of the cocktail was reduced to 5.4 log cfu/mL (2.5 log reduction) indicating high sensitivity of *P. odorifer* to thermal inactivation. After a 5-min holding time at 63°C, 3.5 log cfu/mL of *P. odorifer* remained viable. No further reduction of *P. odorifer* was achieved with additional treatment at 63°C for up to 30 min. Time and temperature combinations of 63°C for 5 to 30 min, 70°C for 0 to 10 min, and 72°C for 15 s did not differ in their lethality toward the *P. odorifer* cocktail, with survivors ranging between 3.0 and 3.1 log cfu/mL. These results indicated presence of a thermally resistant subpopulation of *P. odorifer*. The inoculation preparation procedure (25°C, 24 h) for the thermal inactivation study likely supported initiation of *P. odorifer* sporulation. Additional inoculated milk samples were heat-treated at 80°C for 12 min, demonstrating a spore population of 2.0 log cfu/mL in the milk. Thermal resistance of *Paenibacillus* spores has been recorded in the literature; however, the emphasis has been on spores that display excessive thermal resistance. For example, *Paenibacillus* spores survived heat treatments up to 120°C for 30 s (te Giffel et al., 2002). Furthermore, *Paenibacillus* species have been isolated from UHT-pasteurized milk (280°C, 2 s) (Scheldeman et al., 2004; Deeth, 2017). Overall, our results indicate that vegetative cells of *P. odorifer* are extremely sensitive to heat and would be easily inactivated by HTST pasteurization, even at high cell densities, and thus would not pose an increased spoilage risk for reworked milk. However, due to high thermal resistance of *P. odorifer* spores and perhaps another thermoduric cell type, it is critical to determine whether these thermally resistant subpopulations develop in milk held at refrigeration for extended periods.

**Figure 2:**
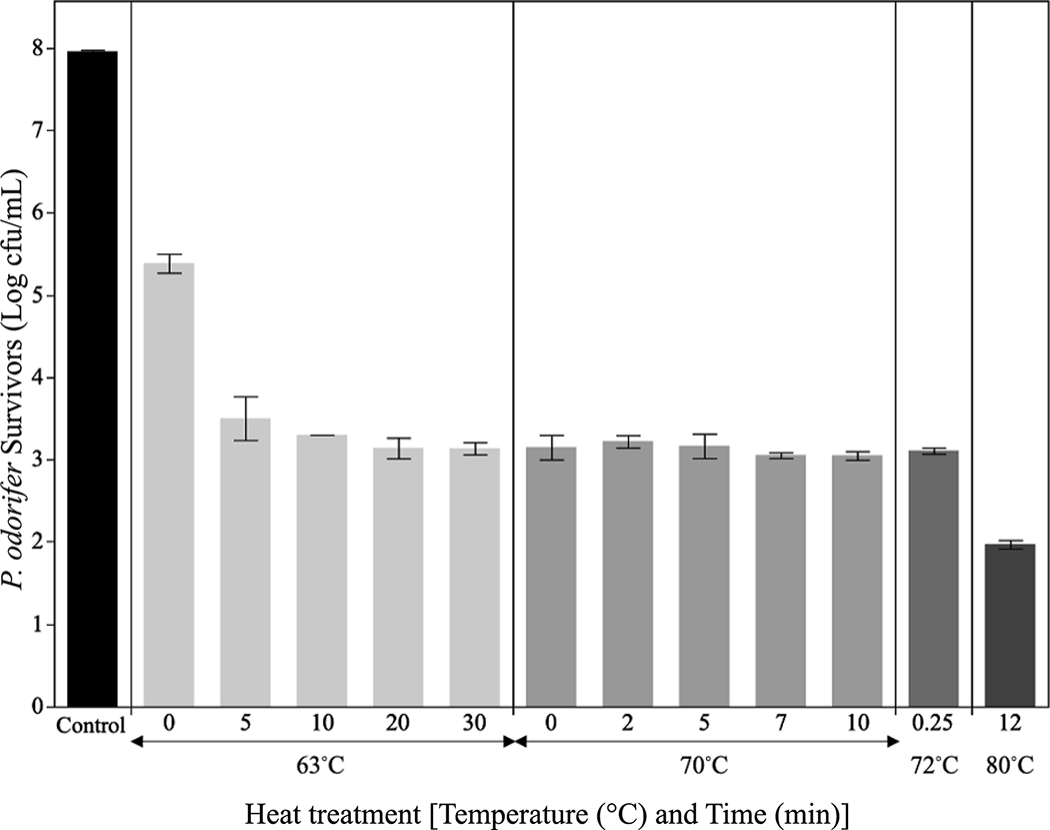
Thermal inactivation of *Paenibacillus odorifer* cocktail grown in pasteurized reduced fat milk at 25°C for 24 h. Data are displayed as mean ± standard error (n = 3). Bars labeled as a treatment time of 0 min represent inactivation due to the come-up time at each temperature. Treatment at 80°C for 12 min is the standard thermal treatment for enumeration of bacterial spores.

A second growth study of *P. odorifer* strains in fluid milk at 4°C was performed to monitor development of thermally resistant subpopulations with extended storage (Figure 3). All *P. odorifer* isolates displayed a similar rapid growth rate (0.40–0.52 log cfu/mL per day) in fluid milk during the first 10 d of storage. However, between 10 and 15 d, no growth was observed for any of the isolates, and cell density stalled at 5.3 to 6.2 log cfu/mL. *Paenibacillus odorifer* transitioned into a second slower growth phase (0.09–0.14 log cfu/ml/d) from 15 d to 25 or 30 d. This growth pattern was not obvious in the previous growth studies at 4°C reported in Figure 1. Further review of the previous data identified a similar pattern for *P. odorifer* growth at 7°C in milk (Figure 1); however, the stall occurred earlier (5–9 d) and at higher cell density (6.3–7.4 log cfu/mL). This diauxic behavior may be linked to depletion of a primary carbon or nitrogen source followed by transition and utilization of a secondary nutrient source (Chu and Barnes, 2016).

**Figure 3:**
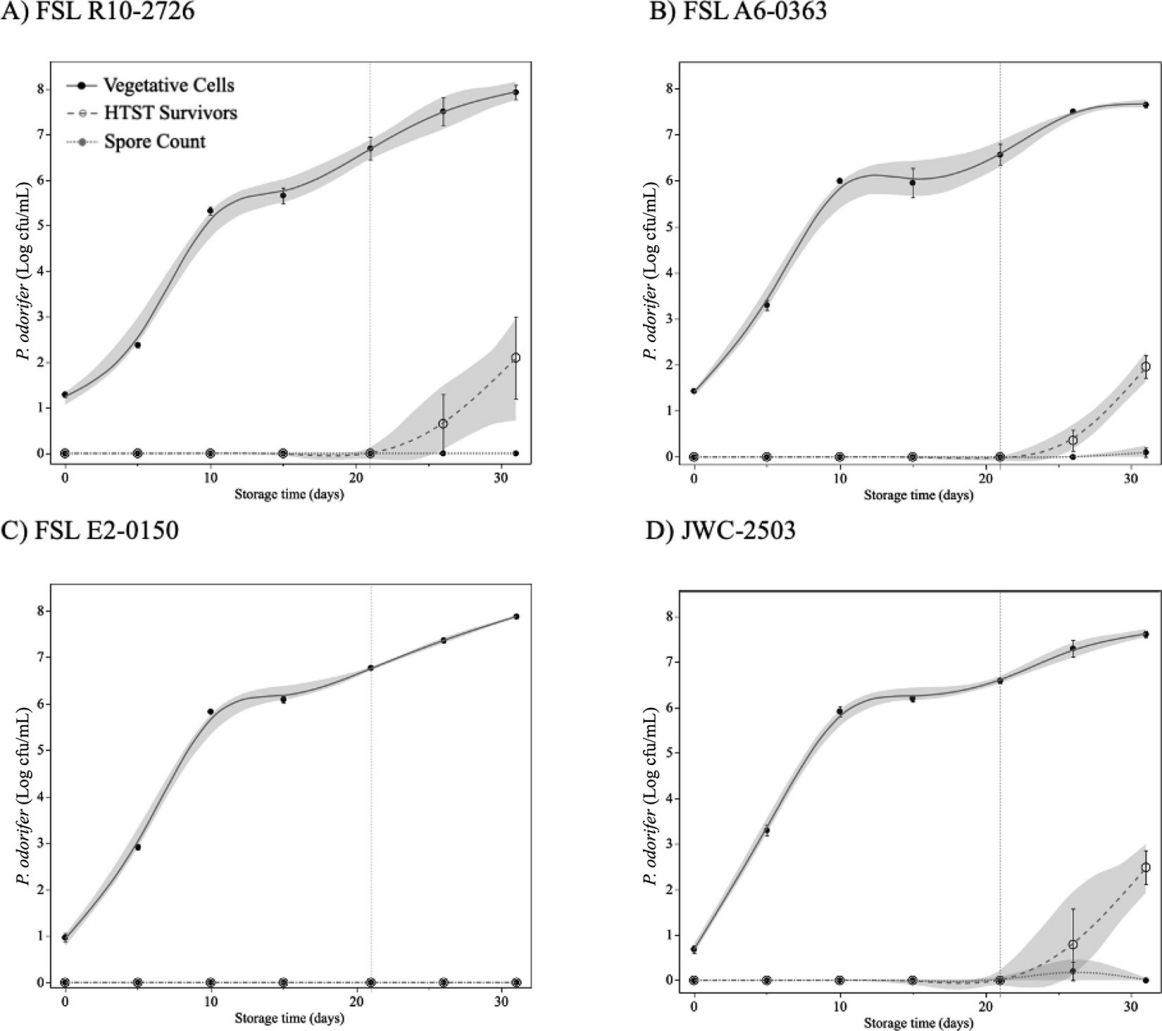
Growth and sporulation of *Paenibacillus odorifer* strains (A) FSL R10-2726, (B) FSL A6-0363, (C) FSL E2-0150, and (D) JWC-2503 in fluid milk (2%) stored at 4°C for 31 d. HTST survivors and spore counts were subpopulations that survived thermal treatments of 72°C for 15 s and 80°C for 12 min, respectively. Data points indicate mean log cfu/mL (n = 3) ± standard error. The dashed line at 21 d indicates the typical end of shelf life and the last potential day to use as rework. Samples with no recovered colonies are reported as equivalent to the detection limit of 0 log cfu/mL (1 cfu/mL).

For the first 21 d of storage in inoculated milk at 4°C, the *P. odorifer* population was highly sensitive to thermal treatment (>6.5 log reduction at 72°C, 15 s) and was presumed to be completely composed of vegetative cells (Figure 3). We continued to incubate samples beyond the 21-d shelf life to determine whether thermally resistant subpopulations could develop under refrigeration conditions. Thermally resistant subpopulations (72°C, 15 s) were first detected at 26 d; however, detection was intermittent across replicates, suggesting a recent change. *Paenibacillus odorifer* FSL R10-2726 had a resistant subpopulation of 1.95 log cfu/mL (vegetative count: 8.00 log cfu/mL) in one sample replicate, whereas JWC-2503 and FSL A6-0363 had low levels of resistant cells (0.3 and 2.4 log cfu/mL) in 2 replicates. With continued storage (31 d), thermally resistant subpopulations of *P. odorifer* were detected in milk samples inoculated with FSL R10-2726, JWC-2503, and FSL A6-0363, ranging between 0.30 and 3.12 log cfu/mL. *Paenibacillus odorifer* FSL E2-1050 did not develop a thermally resistant subpopulation. Data suggest that strains developed resistant subpopulations as they approached their maximum cell density (stationary phase), whereas FSL E2-1050 was still actively replicating (Figure 3). Only one inoculated milk sample (one replicate of FSL A6-0363) produced a single surviving cell (0.3 log cfu/mL) after treatment at 80°C for 12 min (spore count). It is possible that continued incubation at 4°C would have resulted in further development of thermally resistant subpopulations of all strains. Collectively, these results suggest that *P. odorifer* may transition to a cell type with intermediate thermal resistance that is distinct from a true bacterial spore. Research on the sporulation process of *Paenibacillus polymyxa* has identified several subpopulations differing in size and membrane integrity (Comas-Riu and Vives-Rego, 2002). These results also suggest that utilizing the standard thermal treatment for spore counts (Frank and Yousef, 2004) may underestimate populations of *P. odorifer* that could survive pasteurization and affect milk quality. The recommended process for spore enumeration (80°C, 12 min) was based on the thermal resistance of a variety of mesophilic and thermophilic sporeformers (Warth, 1978). No psychrotrophic sporeformers were included in that study, and the authors report a correlation of maximum growth temperature and spore death temperature, suggesting that psychrotrophic sporeformers may be underestimated by this method. Sun et al. (2021) also considered this methodological concern and opted to enumerate *P. odorifer* spores following a thermal treatment of 63°C for 30 min. Additional research on thermal treatments for quantification of psychrotrophic spores or other heat-resistant bacterial subpopulations would facilitate improved understanding of the source and behavior of *Paenibacillus* on farm and during raw milk handling.

The overall objective of our research was to evaluate the potential impact of *P. odorifer* as a spoilage organism in milk containing rework. This study demonstrated that *P. odorifer* is capable of rapid growth and achieves high cell density in both reduced fat and chocolate milk stored at 4°C and 7°C. We demonstrated that vegetative cells of *P. odorifer* are sensitive to pasteurization treatments; however, this species can develop thermal resistant subpopulations. Although these thermal resistant subpopulations can survive pasteurization, we demonstrate that they do not develop in fluid milk samples stored at 4°C for up to 21 d (maximum storage time before rework) and therefore are unlikely reduce the shelf life of milk products containing rework in alignment with the Pasteurized Milk Ordinance (US Food and Drug Administration, 2017). *Paenibacillus odorifer* is unlikely to contribute to the reduced shelf life of fluid and flavored milk products containing rework.
